# Sex-Specific Associations Between Bipolar Disorder Pharmacological Maintenance Therapies and Inpatient Rehospitalizations: A 9-Year Swedish National Registry Study

**DOI:** 10.3389/fpsyt.2020.598946

**Published:** 2020-11-12

**Authors:** Dragos C. Ragazan, Jonas Eberhard, Jonas Berge

**Affiliations:** ^1^Division of Psychiatry, Department of Clinical Sciences Lund, Faculty of Medicine, Lund University, Lund, Sweden; ^2^Vancouver Coastal Health Authority, University of British Columbia Hospital Detwiller Pavilion, Vancouver, BC, Canada; ^3^Region Skåne, Adult Psychiatry Clinic Helsingborg, Helsingborg, Sweden; ^4^Region Skåne, Adult Psychiatry Clinic Malmö, Addiction Centre Malmö, Malmö, Sweden

**Keywords:** bipolar disorder, pharmacoepidemiogy, rehospitalization, sex-based analysis, sex-based differences, register, maintenance therapy

## Abstract

**Background:** Long-term pharmacological maintenance therapy is often essential among people with bipolar disorder to reduce the need for inpatient care. Sex-specific responses to maintenance therapies are expected but remain largely unknown. Here, we examined for sex-specific associations between common maintenance therapies for bipolar disorder with inpatient rehospitalizations following patients' index discharges during 2006–2014.

**Methods:** Population-based data on maintenance therapies and rehospitalizations were extracted from Swedish national registries. We adopted the within-individual design to compare the time on- vs. off- maintenance therapy for males and females, respectively. Extended stratified Cox proportional hazards regression models were employed to quantify the rate of rehospitalization as a function of common maintenance drugs and other important time-varying control variables.

**Results:** Our primary analysis included 22,681 bipolar disorder rehospitalizations by 6,400 males and 9,588 (60.0%) females over an observation time of 62,813 person-years. The time spent on- vs. off- maintenance lithium, lamotrigine, quetiapine, or olanzapine was statistically significant upon adjustment among either sex for reducing the rate of bipolar rehospitalizations. Adjusted sex-specific statistically significant associations were also observed. Among females, the time on- (vs. off-) long-acting injectable risperidone reduced the rate of bipolar rehospitalizations by 73% (56–84%), carbamazepine by 44% (18–62%), aripiprazole by 29% (13–42%), and valproate by 23% (11–33%); whereas among males, ziprasidone by 65% (41–79%).

**Conclusion:** The effectiveness of most maintenance therapies is generally comparable and uniform among both males and females. Despite some statistically significant sex-specific associations, estimates for each drug were fairly consistent between sexes.

## Introduction

Bipolar disorder is a chronic, multiphasic and recurrent psychiatric condition characterized by pronounced, yet variable oscillations in mood, energy, and functioning capacity. Recent meta-analyses have found 35–44% of patients to relapse within the first year of follow-up (1, 2), with a median of 1.4 years between index and subsequent episodes (2). Though the course of illness is indeed heterogenous, impairment and disability may accelerate over time as successive episodes tend to be greater in length or symptom severity, and further increase the risk of recurrence (3). Inpatient treatment is often warranted for acute stabilization whereby pharmacological therapies are typically (re)introduced or optimized. Long-term maintenance therapy may however be deemed essential and continued indefinitely to prevent new episodes even if clinical remission is achieved (4). While lithium is considered as the gold-standard for maintenance therapy, Scandinavian prescription patterns indicate that several other mood-stabilizing antiepileptics (e.g., lamotrigine) and atypical antipsychotics (e.g., quetiapine, olanzapine) have surged in popularity (5, 6). But despite pharmacological advances, responses to treatment remain modest. In a UK population-based cohort study, treatment failure (i.e., monotherapy discontinued, or adjunct therapy introduced) occurred in 75% of patients prescribed lithium, olanzapine, and quetiapine by 2.05, 1.13, and 0.76 years, respectively (7). Though medications are generally well-tolerated (8), non-adherence remains an issue and can prompt rebound symptoms or relapse that may require rehospitalization (9). In Sweden, people with bipolar disorder are twice as likely than the general public to require inpatient care for any reason, psychiatric or otherwise (10). These admissions can therefore act as a robust marker for relapse, and in turn, as a clinically meaningful outcome for maintenance therapy.

Observational studies readily complement randomized controlled trials (RCTs) for investigations on maintenance therapies' efficacy and tolerability. The latter designs have however been criticized for their industry sponsorships, stringent inclusion/exclusion criteria, enriched methodologies (i.e., participants stabilized with the drug(s) under investigation prior to randomization), shortened follow-up periods and high levels of attrition; all which ultimately cast doubt on their generalizability and clinical utility (4, 11, 12). Population-based register studies, especially those with nationwide data that span over several years can therefore be advantageous (11). Still, without randomization procedures, these observational studies could be subject to confounding-by-indication whereby those who present with more severe symptoms might be treated more aggressively but lead to seemingly less favorable outcomes (13). Reliance on time-fixed treatment and other control variables or neglect of medication switches further risk biased estimates (14). Within-individual designs which allow participants to act as their own controls can circumvent these issues (13) (see later). One such study by Joas et al. (15) found that during a 3-year period, the time spent on- vs. off- lithium maintenance reduced the rate of psychiatric rehospitalizations among Swedes with bipolar disorder by 34% (30–38%); valproate by 27% (21–33%), olanzapine by 23% (17–28%), lamotrigine by 22% (16–27%), and quetiapine by 18% (11–24%). Similar findings were later attained in another population-based within-individual study from Finland (16) that also demonstrated the superiority of long-acting injectable (LAI) formulations over their oral counterparts.

A caveat with within-individual designs is that no estimate is derived for any time-fixed variable; therefore, sex-specific differences cannot be examined, unless analyses are stratified by sex. Though encouraged, this approach is seldom practiced in psychiatric research (17). Sex-specific responses to psychopharmacological therapies remain largely unknown but are expected (18–21). The pharmacokinetic profile (i.e., absorption, distribution, metabolism, elimination) of psychotropic drugs can vary by sex and lead to altered serum concentrations impacting their efficacy and tolerability (21). Hormonal changes during the menstrual cycle may also trigger fluctuations in treatment response (20, 22). Unfortunately, females, especially those of childbearing age, have however been historically excluded from drug developments and clinical trials, and consequently, the majority of research and care has been based on male physiology (23).

The influence of sex on bipolar disorder is controversial. Some studies have showed sex-related differences in polarity, symptomology, and comorbidity (24–26); though others have not (27, 28). Prescription patterns could also vary but this too is disputed. For example, Karanti et al. (29) found Swedish males to be significantly more likely to receive outpatient treatment with lithium, and females with lamotrigine; whereas we previously found no sex-specific differences in treatment among Swedes within the 3-months prior to their hospitalizations (30).

There is an urgent need to consider the biological sex (and the broader societal construct of gender) in pharmacoepidemiological studies as to improve our understanding on how sex (and gender) may moderate responses to maintenance therapy. Doing so will enhance our capacity to provide sex- (and gender-) sensitive treatment strategies and maximize chances for long-term recovery. Here, we aim to adopt the within-individual design with national registry data to examine for sex-specific associations between the time spent on- vs. off- maintenance therapy for bipolar disorder with inpatient rehospitalizations following patients' index discharges, during 2006–2014. We hypothesized that the effectiveness of some therapies would vary by sex, but we did not have a priori assumption.

## Methods and Materials

### National Registries and Ethical Considerations

We relied on data extracted from population-based registries maintained by the Swedish National Board of Health and Welfare. In Sweden, residents are assigned unique personal identification numbers which allow for accurate linkages between registries. These identifiers were replaced with arbitrary codes prior access; hence, obtaining patients' informed consent was neither required nor feasible. This study extends another project previously approved by the Regional Board of Ethics, Stockholm (2015/833-31/2).

We retrieved data on inpatient admissions from the National Patient Register (NPR) which achieved full national coverage of all inpatient hospitalizations, circa 1987 (31). This register records patients' age, sex, date of admission and discharge, duration of admission, healthcare department, and diagnoses given at discharge. The latter are now coded according to the International Classification of Diseases and Related Health Problems, Tenth Revision (ICD-10) and are recognized as valid for related epidemiological research (32). We also derived data on maintenance therapies from the Prescribed Drug Register (PDR) which contains information on drugs dispensed in all Swedish ambulatory settings since July 2005, including their Anatomical Therapeutic Chemical (ATC) codes, Defined Daily Doses, and dispensing dates (33). Lastly, we referred to the Cause of Death Register (34) to censor any deceased patients at the date of their death, if such had occurred during our study period.

### Study Cohort

We identified all patients aged ≥18 years with index inpatient admissions secondary to bipolar disorder diagnoses (F31x) during 2006–2014. Patients with index inpatient admissions for single (hypo)manic episodes (F30x) were also included given the high likelihood of an underlying bipolar diagnosis. Since our access to the NPR data dated back to 1987, we were able to verify, and exclude patients with any previous psychiatric inpatient admissions coded with bipolar disorder (and/or single [hypo]manic) diagnoses from 1987 to 2006. Hence, we could be fairly certain that our study cohort consisted of all individuals with index bipolar disorder [and/or single (hypo)manic] inpatient admissions from 2006 to 2014; other than the likely rare occurrences of individuals admitted prior to 1987 without further rehospitalizations until 2006 onward. This was done to limit any selection bias owing to individuals with longstanding bipolar disorder diagnoses and/or exposure to prior psychopharmacological therapies, who would have likely differed than those with more recent index inpatient hospitalizations. Patients with index inpatient admissions that exceeded the end date of the study (31 December 2014) were excluded as they would no longer contribute toward the time at risk for subsequent rehospitalizations.

### Exposure

Our selection of drugs used for maintenance therapy was guided by recommendations from the Swedish Psychiatric Association (35). We collected data on lithium, valproate, lamotrigine, carbamazepine, quetiapine, olanzapine, aripiprazole, risperidone, paliperidone, and ziprasidone. We further included data on clozapine as suggested for the management of refractory symptoms (36). Corresponding ATC codes are issued in the supplementary material (Table e1). We also examined Defined Daily Doses to distinguish between oral and LAI formulations; though this was possible just for risperidone and paliperidone, respectively. Antidepressants were not considered as their role in maintenance therapy remains controversial (4, 36).

We sought to reflect routine clinical practice whereby prescriptions for maintenance therapy are typically written once every 3 months. Exposure to each drug was therefore defined, as done in previous studies (15, 37, 38), using sequences of at least two consecutive prescriptions, each dispensed <3 months (92 days) apart. A maintenance period then started on the date of the first dispensed prescription in a sequence and ended on the date of the last dispensed prescription in that given sequence. Conversely, the time between prescriptions dispensed beyond this 3-month threshold were defined as non-maintenance periods. Another maintenance period was considered to have started again on the date of the next dispensed prescription of the following sequence. One-time dispensed prescriptions were excluded. We used PDR data from 1 July 2005 to 30 September 2015 to determine whether patients were on a maintenance therapy at the start and end of the study.

### Outcome

The primary outcome of this study was rehospitalization for bipolar disorder defined by any ICD-10 related discharge diagnosis (F31x) following patients' index bipolar admissions during the years, 2006–2014. Rehospitalizations for single (hypo)manic episodes (F30x) and for unipolar depressive episodes (F32x, F33x) were also here included. We also considered a broader secondary outcome of rehospitalization owing to any psychiatric reason (Fx). For both outcomes, rehospitalization was to occur at least 1 day after the preceding discharge, and transfers between different hospital departments were classified as a single (re)hospitalization.

### Statistical Analysis

We extended the stratified Cox proportional hazards regression model to allow for the within-individual design and to quantify the associations between the time spent on- vs. off- maintenance therapy with rehospitalizations for males and females, respectively. In this design, each patient is assigned their own unique stratum and serves as their own matched control thereby facilitating comparisons between their time on- and off- maintenance therapy. All probable time-fixed confounders (e.g., genetic predisposition, baseline illness severity) are therefore implicitly controlled for. Only rehospitalized patients who experienced change(s) to their maintenance therapy contributed directly to the estimand; otherwise all patients contributed indirectly by providing information on other important time-varying variables.

Follow-up was divided into consecutive intervals. A new interval commenced after a switch in maintenance therapy [i.e., drug maintenance discontinued or (re)introduced] or following discharge from rehospitalization. With respect to the latter, we restarted the next interval at baseline as to set the underlying time scale as the time since the last rehospitalization. Time spent hospitalized was excluded from follow-up.

All drugs were coded as time-varying dichotomous exposures and added to the models simultaneously. For each outcome we adjusted for other probable time-varying confounders: cumulative number of switches per drug; polypharmacy; number of previous rehospitalizations; age, bipolar subdiagnosis and history of substance- and alcohol- use disorder comorbidity at past admission; duration of past admission; and year of last discharge and refill, respectively. Quadratic functions were further considered to allow for non-linear effects where appropriate. These covariates are defined in the supplementary material (Table e2). Relative comparisons of Akaike information criterion values and concordance indices facilitated model selection (39). Robust standard errors were used to account for correlations among the multiple observations per patient (40). The proportional hazards assumption was graphically inspected (41) and found as satisfied. We provided adjusted hazard ratios (aHRs) and 95% confidence intervals (CIs) for the total sample and for males and females, respectively, to assess for significant sex-specific associations. *P*-values were corrected for multiple comparisons (42) with a significance level set to 0.05. We used *R* version 3.6.1 (43) for all data preparation and analyses.

### Sensitivity Analyses

We varied the definitions of the cohort, exposure and outcome of our primary analysis to form the basis of our sensitivity analyses. (i) We restricted the sample to the incident cohort whereby only patients who were (a) naïve to the maintenance drugs before the start of the study who later (b) experienced variation in their maintenance therapy (≥1 drug switch) and were (c) eventually rehospitalized for at least one bipolar episode were included to account for bias owing to prior therapy. Likewise, (ii) we restricted the sample to patients who only ever received a bipolar diagnosis and excluded those rehospitalized for other psychiatric reasons to account for diagnostic misclassification. With respect to possible exposure misclassification, we also trialed alternative thresholds of (iii) 2- and (iv) 4-months (62 and 122 days, respectively) between dispensed prescriptions to instead define maintenance therapy, and also (v) extended each maintenance period by 30 days to account for potential underexposure. Lastly, (vi) we restricted the outcome to only manic (F30x, F31.0–F31.2), depressive (F31.3–F31.5, F32x, F33x) or mixed (F31.6) episodes to account for the heterogeneity in rehospitalizations coded with multiple diagnoses or with less informative diagnostic clusters (F31.7–F31.9).

## Results

Our study cohort included 6,400 males [mean (SD) age, 44.2 (16.3) years] and 9,588 females [mean (SD) age, 43.1 (17.4) years] with index bipolar disorder hospitalizations during the years, 2006–2014. Collectively, patients were followed upon discharge for a total of 62,813 person-years, with a mean (SD) follow-up time of 3.8 (2.5) and 4.0 (2.5) years for males and females, respectively. Among these patients, 2,851 (44.6%) males and 4,665 (48.7%) females were rehospitalized at least once more with a bipolar disorder related diagnosis (~0.56 rehospitalizations per patient-year); whereas 3,392 (53.0%) males and 5,374 (56.0%) females were rehospitalized at least once more for any psychiatric reason (~0.84 rehospitalizations per patient-year). Cohort characteristics, including information on patients' maintenance therapies are provided in Table 1 with data stratified and compared by sex. Test statistics are outlined in the footnote.

**Table 1:** Characteristics of the patient cohort; stratified and compared by sex (Sweden 2006–2014).

	**Total cohort**	**Males**	**Females**	***p*-value^a^**
Patients followed, *n (%)*	15,988 (100)	6,400 (40.0)	9,588 (60.0)	<0.001
Patients readmitted for bipolar disorder, *n (%)*	7,516 (47.0)	2,851 (44.6)	4,665 (48.7)	<0.001
Patients readmitted for any psychiatric reason*, n (%)*	8,766 (54.8)	3,392 (53.0)	5,374 (56.0)	<0.001
Years of follow-up per patient, *mean (sd)*	3.9 (2.5)	3.8 (2.5)	4.0 (2.5)	<0.001
Died during follow-up*, n (%)*	1,292 (8.1)	642 (10.0)	650 (6.8)	<0.001
Bipolar readmissions per patient-year observed, *mean (sd)*	0.56 (3.98)	0.55 (3.69)	0.57 (4.16)	0.794
Readmissions for any psychiatric reason per patient-year observed, *mean (sd)*	0.84 (4.29)	0.81 (3.95)	0.86 (4.51)	0.484
Age in years at index admission, *mean (sd)*	43.5 (17.0)	44.2 (16.3)	43.1 (17.4)	<0.001
Treated with … at any time during follow up, *n (%)*				
Any of the 13 drugs	14,586 (91.2)	5,708 (89.2)	8,878 (92.6)	<0.001
Lithium	7,977 (49.9)	3,186 (49.8)	4,791 (50.0)	0.821
Valproate	4,052 (25.3)	1,744 (27.3)	2,308 (24.1)	<0.001
Lamotrigine	6,758 (42.3)	2,291 (35.8)	4,285 (46.6)	<0.001
Carbamazepine	719 (4.5)	327 (5.1)	392 (4.1)	0.003
Quetiapine	6,634 (41.5)	2,369 (37.0)	4,265 (44.5)	<0.001
Olanzapine	6,516 (40.8)	2,793 (43.6)	3,723 (38.8)	<0.001
Aripiprazole	2,929 (18.3)	963 (15.0)	1,966 (20.5)	<0.001
Risperidone (oral)	2,143 (13.4)	779 (12.2)	1,364 (14.2)	<0.001
Risperidone (LAI)	267 (1.7)	112 (1.8)	155 (1.6)	0.529
Paliperidone (oral)	166 (1.0)	63 (1.0)	103 (1.1)	0.633
Paliperidone (LAI)	123 (0.8)	54 (0.8)	69 (0.7)	0.406
Ziprasidone	454 (2.8)	103 (1.6)	351 (3.7)	<0.001
Clozapine	236 (1.5)	103 (1.6)	133 (1.4)	0.256
Treated with ≥2 drugs during an observation period, at any time during follow-up, *n (%)*	10,765 (67.3)	4,149 (64.8)	6,616 (69.0)	<0.001
Changed maintenance therapy by study end, *n (%)*	12,903 (80.7)	5,000 (78.1)	7,903 (82.4)	<0.001
History of comorbid… by study end, *n (%)*				
Substance use disorder	2,792 (17.5)	1,241 (19.4)	1,551 (16.2)	<0.001
Alcohol use disorder	3,092 (19.3)	1,552 (24.3)	1,540 (16.1)	<0.001

*sd, standard deviation; LAI, long-acting injectable*.

a *Fisher's exact test used for all categorical variables with the exception for “patients followed” where the chi-square test was used; Welch's two sample t-test used for all continuous variables*.

The majority of males (89.2%) and females (92.6%) were treated at one point with any of the investigated maintenance drugs and about two-thirds of all patients (males 64.8%, females 69.0%) were treated with at least two drugs concomitantly. Approximately 80% of the total cohort (males 78.1%, females 82.4%) changed their maintenance therapy by the end of the study. The most prevalent drugs among all patients were lithium (49.9%), lamotrigine (42.3%), quetiapine (41.5%), olanzapine (40.8%), and valproate (25.3%); and the least common were paliperidone (LAI 0.8%; oral 1.0%), clozapine (1.5%), and LAI risperidone (1.7%). A significantly greater proportion of males were treated at one point with olanzapine (43.6 vs. 38.8%, *p* < 0.001), valproate (27.3 vs. 24.1%, *p* < 0.001), and carbamazepine (5.1 vs. 4.1%, *p* = 0.003); while a significantly greater proportion of females were treated at one point with lamotrigine (46.6 vs. 35.8%, *p* < 0.001), quetiapine (44.5 vs. 37.0%*, p* < 0.001), aripiprazole (20.5 vs. 15.0%, *p* < 0.001), oral risperidone (14.2 vs. 12.2%, *p* < 0.001), and ziprasidone (3.7 vs. 1.6%, *p* < 0.001). The number of patients on maintenance lithium, LAI risperidone, clozapine, or paliperidone (LA, oral) did not significantly vary by sex.

In total, 22,681 bipolar related rehospitalizations occurred during the study period. Table 2 presents the results from our primary within-individual analysis, adjusted for each maintenance drug and the other time-varying probable confounders. With respect to the total cohort, the time spent on- vs. off- maintenance therapy with the following drugs was significantly associated with a reduced rate of rehospitalization for bipolar disorder upon discharge from index admissions: LAI risperidone by 75% (38–90%, *p* = 0.005); lithium by 31% (25–36%, *p* < 0.001); lamotrigine by 29% (23–35%, *p* < 0.001); aripiprazole by 28% (14–39%, *p* < 0.001); olanzapine by 24% (17–30%, *p* < 0.001); quetiapine by 21% (14–28%, *p* < 0.001); and valproate by 17% (8–25%, *p* < 0.001). Sex-specific associations were found whereby the time spent on- vs. off- LAI risperidone (aHR 0.27, 95% CI: 0.16–0.44, *p* < 0.001), carbamazepine (aHR 0.56, 95% CI: 0.38–0.82, *p* = 0.004), aripiprazole (aHR 0.71, 95% CI: 0.58–0.87, *p* = 0.002), and valproate (aHR 0.77, 95% CI: 0.67–0.89, *p* < 0.001) were significant only among females; whereas ziprasidone (aHR 0.35, 95% CI: 0.21–0.59, *p* < 0.001) was significant only for males. Maintenance with lithium, lamotrigine, quetiapine, and olanzapine were significantly associated for both sexes. No sex-specific estimates were possible for paliperidone (oral or LAI) due to infrequent use.

**Table 2:** Adjusted^a^ associations between the time spent on- vs. off- maintenance therapy with bipolar disorder rehospitalization (Sweden, 2006–2014), within-individual design; extended Cox regression model.

**Maintenance drug**	**Total cohort (*n* = 15,988)**		**Males (*n* = 6,400)**		**Females (*n* = 9,588)**	
	**Hazard ratio^a^ (95% CI)**	***p*-value^b^**	**Hazard ratio^a^ (95% CI)**	***p*-value^b^**	**Hazard ratio^a^ (95% CI)**	***p*-value^b^**
Lithium	**0.69 (0.64–0.75)**	**<0.001**	**0.64 (0.57–0.73)**	**<0.001**	**0.71 (0.64–0.78)**	**<0.001**
Valproate	**0.83 (0.75–0.92)**	**<0.001**	0.89 (0.77–1.04)	0.215	**0.77 (0.67–0.89)**	**<0.001**
Lamotrigine	**0.71 (0.65–0.77)**	**<0.001**	**0.72 (0.63–0.83)**	**<0.001**	**0.69 (0.62–0.76)**	**<0.001**
Carbamazepine	0.72 (0.51–1.00)	0.076	1.10 (0.70–1.74)	0.756	**0.56 (0.38–0.82)**	**0.004**
Quetiapine	**0.79 (0.72–0.86)**	**<0.001**	**0.79 (0.68–0.92)**	**0.006**	**0.78 (0.69–0.87)**	**<0.001**
Olanzapine	**0.76 (0.70–0.83)**	**<0.001**	**0.76 (0.67–0.87)**	**<0.001**	**0.74 (0.67–0.83)**	**<0.001**
Aripiprazole	**0.72 (0.61–0.86)**	**<0.001**	0.76 (0.56–1.02)	0.113	**0.71 (0.58–0.87)**	**0.002**
Risperidone (oral)	0.93 (0.78–1.11)	0.495	0.92 (0.67–1.28)	0.734	0.94 (0.76–1.16)	0.609
Risperidone (LAI)	**0.25 (0.10–0.62)**	**0.005**	0.27 (0.02–3.17)	0.368	**0.27 (0.16–0.44)**	**<0.001**
Ziprasidone	0.77 (0.48–1.22)	0.328	**0.35 (0.21–0.59)**	**<0.001**	0.96 (0.62–1.47)	0.854
Clozapine	0.64 (0.37–1.11)	0.158	0.53 (0.18–1.62)	0.343	0.61 (0.36–1.02)	0.084
Events, *n*	22,681		8,338		14,343	

*CI, confidence interval; LAI, long-acting injectable*.

a *Adjusted for: each other drug [including paliperidone (oral and LAI)]; cumulative number of switches per drug; polypharmacy; number of previous bipolar rehospitalizations; age, subdiagnosis, and history of substance- and alcohol- use disorder comorbidity at past admission; duration of past admission; year of last discharge and refill, respectively*.

b *Corrected for multiple comparisons with the Benjamini–Hochberg procedure*.

*Bold values refer to statistically significant associations (p <0.05 post Benjamini-Hochberg procedure)*.

Table 3 displays the results from our secondary within-individual analysis with rehospitalization owing to any psychiatric reason (*N* = 35,584) as the outcome. Sex-specific associations were also found whereby the time spent on- vs. off- carbamazepine (aHR 0.57, 95% CI: 0.44–0.76, *p* <0.001), clozapine (aHR 0.67, 95% CI: 0.49–0.91, *p* = 0.016), aripiprazole (aHR 0.80, 95% CI: 0.71–0.91, *p* <0.001), and oral risperidone (aHR 0.83, 95% CI: 0.71–0.97, *p* = 0.032) were only significant among females; and LAI paliperidone (aHR 0.59, 95% CI: 0.12–0.85, *p* = 0.043) was only significant among males. Ziprasidone was no longer exclusively significant among males following the adjustment for multiple comparisons. No other statistically significant sex-related differences were found nor were sex-specific estimates possible for oral paliperidone.

**Table 3:** Adjusted^a^ associations between the time spent on- vs. off- maintenance therapy with psychiatric rehospitalization (Sweden, 2006–2014), within-individual design; extended Cox regression model.

**Maintenance drug**	**Total cohort (n=15,988)**		**Males (n=6,400)**		**Females (n=9,588)**	
	**Hazard ratio^a^ (95% CI)**	***p*-value^b^**	**Hazard ratio^a^ (95% CI)**	***p*-value^b^**	**Hazard ratio^a^ (95% CI)**	***p*-value^b^**
Lithium	**0.69 (0.65–0.74)**	**<0.001**	**0.68 (0.61–0.76)**	**<0.001**	**0.71 (0.65–0.77)**	**<0.001**
Valproate	**0.80 (0.75–0.86)**	**<0.001**	**0.85 (0.75–0.96)**	**0.018**	**0.76 (0.68–0.84)**	**<0.001**
Lamotrigine	**0.72 (0.67–0.77)**	**<0.001**	**0.79 (0.70–0.89)**	**<0.001**	**0.69 (0.64–0.76)**	**<0.001**
Carbamazepine	**0.70 (0.57–0.84)**	**<0.001**	0.86 (0.64–1.14)	0.397	**0.57 (0.44–0.76)**	**<0.001**
Quetiapine	**0.79 (0.74–0.85)**	**<0.001**	**0.77 (0.69–0.87)**	**<0.001**	**0.81 (0.75–0.88)**	**<0.001**
Olanzapine	**0.74 (0.69–0.79)**	**<0.001**	**0.72 (0.64–0.80)**	**<0.001**	**0.75 (0.69–0.82)**	**<0.001**
Aripiprazole	**0.81 (0.73–0.90)**	**<0.001**	0.87 (0.72–1.05)	0.205	**0.80 (0.71–0.91)**	**<0.001**
Risperidone (oral)	**0.83 (0.73–0.94)**	**0.005**	0.81 (0.66–1.00)	0.077	**0.83 (0.71–0.97)**	**0.032**
Risperidone (LAI)	**0.50 (0.38–0.66)**	**<0.001**	**0.52 (0.34–0.79)**	**0.006**	**0.50 (0.36–0.71)**	**<0.001**
Paliperidone (LAI)	**0.60 (0.37–0.97)**	**0.049**	**0.33 (0.12–0.85)**	**0.043**	0.73 (0.38–1.42)	0.427
Ziprasidone	0.82 (0.58–1.17)	0.332	0.59 (0.36–0.97)	0.066	1.02 (0.69–1.51)	0.969
Clozapine	**0.68 (0.51–0.91)**	**0.013**	0.89 (0.40–1.98)	0.793	**0.67 (0.49–0.91)**	**0.016**
Events, *n*	35,584		13,152		22,432	

*CI, confidence interval; LAI, long-acting injectable*.

a *Adjusted for: each other drug (including oral paliperidone); cumulative number of switches per drug; polypharmacy; number of previous psychiatric rehospitalizations; age, subdiagnosis, and history of substance- and alcohol- use disorder comorbidity at past admission; duration of past admission; year of last discharge and refill, respectively*.

b *Corrected for multiple comparisons with the Benjamini–Hochberg procedure*.

*Bold values refer to statistically significant associations (p <0.05 post Benjamini-Hochberg procedure)*.

Results from our sensitivity analyses are issued in the supplementary material (Tables e3–e8) and generally support the associations derived from our primary analysis, notwithstanding some exceptions. No significant sex-specific associations were observed once the sample was restricted to the incident cohort (Table e3), with lithium as the lone statistically significant drug among both sexes. Excluding patients with non-bipolar related psychiatric rehospitalizations (Table e4) showed that quetiapine was significant only among males; otherwise among both groups were valproate and aripiprazole significant, whereas LAI risperidone was non-significant. Upon lowering the threshold between dispensed prescriptions to 2-months (Table e5), valproate, LAI risperidone and ziprasidone were non-significant among either sex, but quetiapine was significant only among females. Increasing the threshold to 4-months (Table e6) led to statistical significance for valproate and aripiprazole among both sexes. Extending each maintenance period by 30 days (Table e7) showed quetiapine to be significant only among females. Finally, restricting our outcome to depressive, manic, or mixed episodes (Table e8) had quetiapine and clozapine reach statistical significance among only females with no male-specific statistically significant associations; aripiprazole, but neither carbamazepine nor LAI risperidone were significant among either sex.

## Discussion

To our knowledge, this is the first study to explicitly examine for sex-specific associations between bipolar disorder maintenance therapies and inpatient rehospitalizations. Our main findings indicate that while prescription trends may follow sex-specific patterns, the effectiveness of most maintenance drugs is generally comparable and uniform among both males and females. Despite some (statistically significant) sex-specific associations, estimates were fairly consistent across drugs and the substantial overlap of their confidence intervals offer no support for sex as an effect modifier.

The most prevalent maintenance therapies: lithium, lamotrigine, quetiapine, olanzapine, and valproate were significantly associated with reduced rates of rehospitalization among either sex. These associations reflect those previously reported in other population-based within-individual studies (15, 16) and complement findings from meta-analyses of RCTs (12, 44, 45). However, valproate was statistically significant only among females when bipolar disorder (cf. psychiatric) rehospitalizations were considered or when maintenance periods were extended by 30-days. Given its established teratogenic potential and risk for polycystic ovary syndrome or other menstrual irregularities (46), it might be that valproate was reserved for females with more severe presentations, who in turn, would have had a greater opportunity to respond to treatment. Still, it was surprising that nearly a quarter of females in this present study were at one point on valproate maintenance. Some might have been unnecessarily at risk for iatrogenic morbidity. Hormonal contraceptives have also been suggested to reduce valproate clearance (18) thereby risking toxicity. Vigilance must be exerted when prescribing valproate to females of reproductive age and routine monitoring of serum concentrations is recommended.

Quetiapine likewise showed inconsistent results in our sensitivity analyses. The association was statistically significant only among males (upon correction for multiple comparisons) when patients with other psychiatric rehospitalizations were excluded, but only among females when maintenance periods were either extended by 30-days or defined by a 2-month threshold (upon said correction), and when rehospitalizations were restricted to manic, depressive or mixed episodes. It is unknown whether responses to quetiapine are sex-specific, though dose-adjusted serum concentrations could be 20–30% higher in females (47). Quetiapine also exists in either immediate- or extended- release formulations. In Sweden, the latter is more often prescribed to those with a higher disease burden or psychiatric comorbidity (48). Therefore, those treated with the extended release formulation could represent a subgroup that is more likely to require inpatient care. We were however unable to consider the prescribed dose or formulation of quetiapine. Further sex-based inquiries on quetiapine are needed to clarify mechanisms, if any, that could optimize treatment response.

Lithium showed one of the strongest effects among all oral therapies whereby the time spent on (cf. off) lithium maintenance reduced the rate of bipolar rehospitalizations by 36% (27–43%) among males, and by 29% (22–36%) among females. The slightly lower latter estimate may reflect a weaker response as seen in a Danish population-based study where females (cf. males) were significantly associated with a 12% (4–21%) greater hazard for lithium “non-response” (i.e., prescribed polypharmacy or rehospitalized) (49). However, a recent meta-analysis that included the aforementioned study and 16 others found no support for sex as a predictor of lithium response (50). Definitions of “response” (or “relapse”) can therefore be variable underscoring the need for more rigorous outcome measures. Nevertheless, sex-specific reasons for stopping lithium have been documented. Öhlund and colleagues found females to be three- and five- times as likely to discontinue lithium due to weight gain and oedema, respectively; whereas males were three-times as likely because they perceived to be well enough without medication, and twice as likely to stop without first consulting their physicians (51). Iatrogenic symptoms ought to be better managed and continuous psychoeducation needs to be provided to promote adherence. It is also important to note that only lithium reached statistical significance in the sensitivity analysis restricted to our incident, drug-naïve cohort [*males* aHR: 0.68, 95% CI: 0.58–0.81, *p* <0.001; *females*: aHR 0.80, 95% CI: 0.68–0.94, *p* = 0.033] thereby emphasizing its potential benefit among patients with index bipolar episodes. In accordance with reviews of observational studies (11) and RCTs (44), we recommend lithium as a first-line agent for the long-term management of bipolar disorder, especially among drug-naïve individuals.

We found LAI formulations to correspond to the strongest effect of reducing either bipolar or psychiatric rehospitalizations. In particular, the time spent on- vs. off- LAI risperidone was significantly associated with a 73% (56–84%) reduced hazard for bipolar rehospitalizations among females; and LAI paliperidone was significantly associated with a 67% (15–88%) reduced hazard for psychiatric rehospitalizations among males. Similarly, Lähteenvuo and colleagues found LAIs (cf. oral formulations) to be significantly associated with a 30% (10–45%) reduced hazard for psychiatric rehospitalizations; though it was questioned whether findings would generalize to LAI-naïve patients (16). In this present study, <2% of females, were at one point, under maintenance LAI risperidone, and <1% of males were on LAI paliperidone. The current evidence to support the use of LAIs in bipolar disorder is very limited. No significant difference between LAIs and their oral counterparts were seen with respect to relapse, discontinuation, extrapyramidal symptoms nor weight gain in another recent meta-analysis (52). Still, LAIs could be beneficial for those with seldom, yet serious multiple episodes or with low adherence (53).

Statistically significant sex-specific associations were observed with respect to some of the lesser used maintenance therapies, yet differences here might rather be due to reduced statistical power than to true clinically meaningful sex-related differences. We found aripiprazole to be significant only among females for reducing the rate of either bipolar or psychiatric rehospitalizations; though in our sensitivity analyses with patients with non-bipolar rehospitalizations excluded or with rehospitalizations restricted to manic, depressive, or mixed episodes, aripiprazole reached significance among either sex. These findings are then in line with another RCT meta-analysis which found no significant sex-related differences for aripiprazole in preventing manic or depressive relapses (54). Aripiprazole has been suggested as an adjunct for the management of comorbid borderline personality disorder (36); a characteristic seen more often among females (27, 30). It is plausible that aripiprazole was used for similar indications among our cohort (i.e., other comorbidities), though this was not assessed in the present study and merits further investigation. Carbamazepine has also been commonly used for the maintenance of rapid cycling (55); another characteristic seen more often among females (25, 27). This could partly explain the observed female-specific significant associations for carbamazepine in reducing the rate of either bipolar or psychiatric rehospitalizations. We observed a stronger effect for carbamazepine than did Joas et al. (15) (i.e., 30 vs. 8% reduced rate of psychiatric rehospitalization) when we consider the estimate for our total cohort, but only our association was statistically significant. This possibly reflects differences in cohort selection (i.e., we excluded individuals with admissions for bipolar disorder prior to the year 2006), study duration (nine vs. 3 years), and our control for additional time-varying covariates. In any case, carbamazepine was sparingly used among females (or males) in this present study; reflective of its high teratogenic risk and limited demonstrated efficacy (36). Ziprasidone maintenance was also infrequent, though more prevalent among females, presumably due to its lower risk of weight gain as compared to other antipsychotics (56)—a concern often greater for females than males (51). We however found ziprasidone to significantly reduce the rate of bipolar rehospitalizations among males only. This finding does elude us as its pharmacokinetic profile does not appear to vary by sex (57). Oral risperidone displayed a significant female-specific association for reducing the rate of psychiatric but not bipolar rehospitalizations suggesting that the agent was possibly used to manage other non-affective symptoms, though this was not formally evaluated. Our sensitivity analyses however offered no support for this association and its efficacy for treating mixed bipolar symptoms has been shown to be independent of sex (58). Though rarely used, similar significant female-specific associations were seen for clozapine. Sex-specific responses to clozapine however remain inconclusive and understudied (59).

The strengths of this present study include the use of population-based registries to account for all inpatient psychiatric rehospitalizations and commonly prescribed maintenance therapies (i.e., 13 different formulations) among patients first admitted for bipolar disorder in Sweden over a 9-year period. We also adopted the within-individual design to allow for comparisons between the time spent on- vs. off- maintenance therapies and thereby minimized bias owing to confounding-by-indication or to unmeasured time-fixed confounders. Moreover, we controlled for treatment switches in addition to a host of other time-varying confounders, and further performed several sensitivity analyses to test the rigor of our findings.

Limitations are to be acknowledged. Exposure to each maintenance therapy was derived from data on dispensed prescriptions; consequently, adherence might have varied across drugs and/or sex. We therefore relied on sequences of dispensed prescriptions to define our time-dependent maintenance periods. It would have been unlikely for further prescriptions to be retrieved if adherence was an issue. We also trialed thresholds of 2- and 4- months between prescriptions in case dispensing histories deviated from the typical 3-month standard, yet findings were similar. There is the possibility of underexposure as maintenance periods here ended on the date of the last dispensed prescription in a sequence (i.e., patients could have actually been medicated thereafter). This could result in an underestimation of our reported associations, but we also extended each maintenance period by 30-days and reached similar conclusions. Still, we had no data on the prescribed dose(s) nor did we examine combinations of maintenance therapies. Although we controlled for concomitant therapies and polypharmacy, synergistic and/or dose-dependent relationships might have been missed. Likewise, we were unable to consider other pharmacological or psychosocial interventions that could have influenced the need for inpatient care.

Rehospitalizations may not be necessarily interchangeable with relapse. Our investigation was limited to patients ill enough to require inpatient treatment and may not generalize to those treated only as outpatients or with subsyndromal episodes. Also, our results could favor maintenance therapies with greater antimanic or antisuicidal (than antidepressive) properties as inpatient care could be more indicated for those who are manic or at risk for suicidality than those presenting with depressive complaints. Diagnostic uncertainty is another limitation given the overlapping presentations of bipolar disorder with other psychiatric conditions.

We further cannot exclude the possibility of residual time-varying confounding (e.g., rapid cycling; changing symptom severity or psychosocial circumstances). This precludes direct causal inference. Importantly, we might have also been underpowered to accurately assess for sex-specific associations among the lesser prevalent therapies; hence, we cannot accept our hypothesis with full confidence.

In conclusion, we demonstrated that the effectiveness of most maintenance therapies for bipolar disorder is generally well comparable between males and females for reducing the rate of inpatient rehospitalizations, though some lesser used therapies could follow sex-specific trends. Clinicians should adopt sex- and gender- sensitive strategies to ensure the needs and goals of patients are appropriately met. Sex- and gender- based inquiries have long been underrepresented in psychiatric research; seldom have investigators gone beyond control for sex or gender (17). We thereby call upon for more sex- and gender- based psychiatric research to ensure services are rightfully equal and equitable.

## Data Availability Statement

The data analyzed in this study is subject to the following licenses/restrictions: Access to registry data is restricted under the conditions set forth by the Swedish National Board of Health and Welfare. Requests to access these datasets should be directed to the Swedish National Board of Health and Welfare, socialstyrelsen@socialstyrelsen.se.

## Ethics Statement

The studies involving human participants were reviewed and approved by the Regional Board of Ethics, Stockholm (2015/833-31/2). Written informed consent for participation was not required for this study in accordance with the national legislation and the institutional requirements.

## Author Contributions

DCR: conceptualization, methodology, formal analysis, writing—original draft, and writing—review and editing. JE: supervision, funding acquisition, and writing—review and editing. JB: conceptualization, supervision, funding acquisition, and writing—review and editing. All authors contributed to the article and approved the submitted version.

## Conflict of Interest

The authors declare that the research was conducted in the absence of any commercial or financial relationships that could be construed as a potential conflict of interest.
