# Supplementary material for: Sex-Specific Associations Between Bipolar Disorder Pharmacological Maintenance Therapies and Inpatient Rehospitalizations: A 9-Year Swedish National Registry Study
Source: Front Psychiatry. 2020 Nov 12;11:598946. doi: 10.3389/fpsyt.2020.598946 (PMC7688467; doi:10.3389/fpsyt.2020.598946)
Supplement: Supplementary file 1 [file Data_Sheet_1.docx]

Supplementary Material

Table of Contents

[1 Table e1. Anatomical Therapeutic Chemical (ATC) codes for the study drugs used for maintenance therapy in bipolar disorder. 2](#_Toc49250186)

[2 Table e2. Definition of adjusted covariates in the (within-individual) stratified Cox proportional hazards models by the two study outcomes. 3](#_Toc49250187)

[3 Table e3. Sensitivity analysis. Incident cohort.^a^ Adjusted associations between the time spent on- versus off- maintenance therapy with bipolar disorder rehospitalization. Within-individual design; extended Cox regression model. 4](#_Toc49250188)

[4 Table e4. Sensitivity analysis. Patients with non-bipolar related psychiatric rehospitalizations excluded. Adjusted associations between the time spent on- versus off- maintenance therapy with bipolar disorder rehospitalization. Within-individual design; extended Cox regression model. 5](#_Toc49250189)

[5 Table e5. Sensitivity analysis. Alternative threshold of two months (62 days) between dispensed prescriptions to define maintenance therapy. Adjusted associations between the time spent on- versus off- maintenance therapy with bipolar disorder rehospitalization. Within-individual design; extended Cox regression model. 6](#_Toc49250190)

[6 Table e6. Sensitivity analysis. Alternative threshold of four months (122 days) between dispensed prescriptions to define maintenance therapy. Adjusted associations between the time spent on- versus off- maintenance therapy with bipolar disorder rehospitalization. Within-individual design; extended Cox regression model. 7](#_Toc49250191)

[7 Table e7. Sensitivity analysis. Maintenance periods extended by 30 days. Adjusted associations between the time spent on- versus off- maintenance therapy with bipolar disorder rehospitalization. Within-individual design; extended Cox regression model. 8](#_Toc49250192)

[8 Table e8. Sensitivity analysis. Outcome restricted to rehospitalizations for manic, depressive or mixed episodes ^a^. Adjusted associations between the time spent on- versus off- maintenance therapy with bipolar disorder rehospitalization. Within-individual design; extended Cox regression model. 9](#_Toc49250193)

# Table e1. Anatomical Therapeutic Chemical (ATC) codes for the study drugs used for maintenance therapy in bipolar disorder.

| Study drug | ATC code |
| --- | --- |
| Lithium | N05AN01 |
| Valproate | N03AG01 |
| Lamotrigine | N03AX09 |
| Carbamazepine | N03AF01 |
| Quetiapine | N05AH04 |
| Olanzapine | N05AH03 |
| Aripiprazole | N05AX12 |
| Risperidone | N05AX08 |
| Paliperidone | N05AX13 |
| Ziprasidone | N05AE04 |
| Clozapine | N05AH02 |

# Table e2. Definition of adjusted covariates in the (within-individual) stratified Cox proportional hazards models by the two study outcomes.

| Adjusting covariates | 1˚ outcome – bipolar rehospitalization | 2˚ outcome – psychiatric rehospitalization | |
| --- | --- | --- | --- |
| Concurrent use of the other maintenance therapy drugs | Dichotomous; (yes/no) for each drug separately | | |
| Cumulative number of switches per drug since the start of follow-up | 4-level categorical variable (0, 1, 2, ≥3 switches) | | |
| Polypharmacy; treated with ≥2 drugs during the same observation period | Dichotomous; (yes/no) | | |
| Number of previous (bipolar/psychiatric) rehospitalizations | Number of previous *bipolar* rehospitalizations; continuous + quadratic ^a^ | | Number of previous *psychiatric* rehospitalizations; continuous + quadratic ^a^ |
| Age at past admission | Continuous; quadratic function trialed though relative AIC and concordance statistic were not favourable | | |
| Subdiagnosis at past admission | 5-level categorical variable ^b^ (1 = depressive [F31.3-F31.5, F32x, F33x], 2 = manic [F30x, F31.0-F31.2], 3 = mixed [F31.6], 4 = grand ‘other’ [F31.7-F31.9] and if multiple bipolar diagnoses were present for the same admission, 5 = non-bipolar [sub]diagnosis) | | |
| Year of discharge from past admission | Recoded as 1 = 2006, … 9 = 2014. Continuous + quadratic ^a^ | | |
| Duration (i.e., days) of last admission | Continuous + quadratic ^a^; trialed 4-level categorical variable (based on quartiles per calendar year of discharge) though relative AIC and concordance indices were not favourable | | |
| Year of last refill | Recoded as 1 = 2006, … 9 = 2014. Continuous + quadratic ^a^ | | |
| History of substance-use comorbidity at past admission | Dichotomous ^b^ (1 = yes [F11x-F16x, F17-F18x], 0 = none). Diagnosis carried forward for all subsequent observations since the admission where present. | | |
| History of alcohol-use comorbidity at past admission | Dichotomous ^b^ (1 = yes [F10x], 0 = none). Diagnosis carried forward for all subsequent observations since the admission where present. | | |

^a^ Quadratic function decreased Akaike information criterion (AIC) and increased concordance index as compared to model without the quadratic function.

^b^ Codes refer to the ICD-10 (International Classification of Diseases and Related Health Problems, 10^th^ Revision) diagnostic clusters

# Table e3. Sensitivity analysis. Incident cohort^a^. Adjusted associations between the time spent on- versus off- maintenance therapy with bipolar disorder rehospitalization. Within-individual design; extended Cox regression model.

| **Maintenance drug** | **Total cohort (n=2,461)** |  | **Males (n=960)** |  | **Females (n=1,501)** |  |
| --- | --- | --- | --- | --- | --- | --- |
|  | **Hazard ratio^b^ (95% CI)** | **P-value^c^** | **Hazard ratio^b^ (95% CI)** | **P-value^c^** | **Hazard ratio^b^ (95% CI)** | **P-value^c^** |
| **Lithium** | **0.74 (0.66 – 0.84)** | **<.001** | **0.68 (0.58 – 0.81)** | **<.001** | **0.80 (0.68 – 0.94)** | **.033** |
| **Valproate** | 0.89 (0.78 – 1.02) | .323 | 1.00 (0.81 – 1.23) | .998 | 0.82 (0.69 – 0.97) | .083 |
| **Lamotrigine** | 0.85 (0.74 – 0.97) | .123 | 0.87 (0.71 – 1.08) | .406 | 0.84 (0.71 – 1.00) | .158 |
| **Carbamazepine** | 0.83 (0.56 – 1.21) | .526 | 1.13 (0.72 – 1.75) | .812 | 0.60 (0.34 – 1.06) | .232 |
| **Quetiapine** | 0.93 (0.82 – 1.05) | .470 | 0.94 (0.77 – 1.15) | .778 | 0.92 (0.79 – 1.08) | .474 |
| **Olanzapine** | 0.91 (0.80 – 1.04) | .397 | 0.93 (0.75 – 1.14) | .691 | 0.89 (0.75 – 1.06) | .369 |
| **Aripiprazole** | 0.83 (0.62 – 1.11) | .439 | 0.64 (0.39 – 1.05) | .215 | 0.92 (0.68 – 1.25) | .734 |
| **Risperidone (oral)** | 0.95 (0.74 – 1.23) | .845 | 1.07 (0.70 – 1.63) | .906 | 0.92 (0.67 – 1.28) | .734 |
| **Risperidone (LAI)** | 0.57 (0.33 – 0.98) | .214 | 0.64 (0.35 – 1.17) | .334 | 0.89 (0.53 – 1.48) | .734 |
| **Paliperidone (oral)** | 1.50 (0.75 – 3.01) | .470 | 0.95 (0.18 – 4.98) | .989 | nil estimate | – |
| **Paliperidone (LAI)** | 1.12 (0.50 – 2.51) | .874 | nil estimate | – | 1.34 (0.57 – 3.11) | .682 |
| **Ziprasidone** | 1.35 (0.40 – 4.50) | .778 | nil estimate | – | 1.30 (0.37 – 4.59) | .765 |
| **Clozapine** | 1.69 (0.53 – 5.38) | .571 | **0.37 (0.29 – 0.48)** | **<.001** | nil estimate | – |
| **Events, n** | 9,657 |  | 3,790 |  | 5,867 |  |

Abbreviations: *n*=number of; CI = confidence interval; LAI = long-acting injectable

^a^ Defined as patients who were *(a)* naïve to the maintenance drugs before the start of the study who later *(b)* experienced variation in their maintenance therapy (≥1 drug switch) and were *(c)* eventually readmitted for at least one bipolar episode

^b^ Adjusted for: each other drug; cumulative number of switches per drug; polypharmacy; number of previous bipolar rehospitalizations; age, subdiagnosis, and history of substance- and alcohol- use disorder comorbidity at past admission; duration of past admission; year of last discharge and refill, respectively.

^c^ Corrected for multiple comparisons with the Benjamini-Hochberg procedure

# Table e4. Sensitivity analysis. Patients with non-bipolar related psychiatric rehospitalizations excluded. Adjusted associations between the time spent on- versus off- maintenance therapy with bipolar disorder rehospitalization. Within-individual design; extended Cox regression model.

| **Maintenance drug** | **Total cohort (n=12,377)** |  | **Males (n=4,974)** |  | **Females (n=7,403)** |  |
| --- | --- | --- | --- | --- | --- | --- |
|  | **Hazard ratio^a^ (95% CI)** | **P-value^b^** | **Hazard ratio^a^ (95% CI)** | **P-value^b^** | **Hazard ratio^a^ (95% CI)** | **P-value^b^** |
| **Lithium** | **0.66 (0.60 – 0.73)** | **<.001** | **0.65 (0.55 – 0.76)** | **<.001** | **0.67 (0.59 – 0.75)** | **<.001** |
| **Valproate** | **0.78 (0.67 – 0.90)** | **.002** | **0.77 (0.62 – 0.97)** | **.042** | **0.78 (0.64 – 0.94)** | **.017** |
| **Lamotrigine** | **0.67 (0.59 – 0.75)** | **<.001** | **0.67 (0.54 – 0.84)** | **.001** | **0.66 (0.57 – 0.76)** | **<.001** |
| **Carbamazepine** | **0.46 (0.29 – 0.73)** | **.002** | 0.38 (0.12 – 1.20) | .133 | **0.44 (0.24 – 0.79)** | **.010** |
| **Quetiapine** | **0.79 (0.69 – 0.91)** | **.002** | **0.72 (0.58 – 0.89)** | **.007** | 0.84 (0.70 – 0.99) | .060 |
| **Olanzapine** | **0.73 (0.64 – 0.82)** | **<.001** | **0.73 (0.60 – 0.89)** | **.004** | **0.71 (0.61 – 0.83)** | **<.001** |
| **Aripiprazole** | **0.62 (0.48 – 0.79)** | **<.001** | **0.67 (0.47 – 0.95)** | **.038** | **0.55 (0.39 – 0.77)** | **.001** |
| **Risperidone (oral)** | 0.99 (0.76 – 1.29) | .959 | 1.30 (0.79 – 2.14) | .345 | 0.94 (0.70 – 1.27) | .728 |
| **Risperidone (LAI)** | **0.10 (0.02 – 0.66)** | **.028** | 0.07 (0.01 – 0.87) | .054 | 0.16 (0.02 – 1.63) | .163 |
| **Paliperidone (oral)** | 1.24 (0.23 – 6.69) | .851 | 0.63 (0.17 – 2.31) | .533 | nil estimate | – |
| **Paliperidone (LAI)** | 0.71 (0.28 – 1.81) | .530 | nil estimate | – | nil estimate | – |
| **Ziprasidone** | 1.47 (0.84 – 2.57) | .234 | nil estimate | – | 1.42 (0.82 – 2.45) | .254 |
| **Clozapine** | 0.43 (0.15 – 1.25) | .163 | 0.35 (0.09 – 1.39) | .170 | 0.20 (0.03 – 1.35) | .137 |
| **Events, n** | 12,826 |  | 4,746 |  | 8,080 |  |

Abbreviations: *n*=number of; CI = confidence interval; LAI = long-acting injectable

^a^ Adjusted for: each other drug; cumulative number of switches per drug; polypharmacy; number of previous bipolar rehospitalizations; age, subdiagnosis (recoded as a four-level factor as non-bipolar diagnosis level excluded), history of substance- and alcohol- use disorder comorbidity at past admission; duration of past admission; year of last discharge and refill, respectively.

^b^ Corrected for multiple comparisons with the Benjamini-Hochberg procedure

# Table e5. Sensitivity analysis. Alternative threshold of two months (62 days) between dispensed prescriptions to define maintenance therapy. Adjusted associations between the time spent on- versus off- maintenance therapy with bipolar disorder rehospitalization. Within-individual design; extended Cox regression model.

| **Maintenance drug** | **Total cohort (n=15,988)** |  | **Males (n=6,400)** |  | **Female s (n=9,588)** |  |
| --- | --- | --- | --- | --- | --- | --- |
|  | **Hazard ratio^a^ (95% CI)** | **P-value^b^** | **Hazard ratio^a^ (95% CI)** | **P-value^b^** | **Hazard ratio^a^ (95% CI)** | **P-value^b^** |
| **Lithium** | **0.70 (0.65 – 0.76)** | **<.001** | **0.68 (0.60 – 0.77)** | **<.001** | **0.71 (0.64 – 0.78)** | **<.001** |
| **Valproate** | 0.89 (0.80 – 0.99) | .062 | 0.88 (0.75 – 1.04) | .207 | 0.87 (0.75 – 1.01) | .121 |
| **Lamotrigine** | **0.75 (0.70 – 0.82)** | **<.001** | **0.83 (0.72 – 0.95)** | **.019** | **0.71 (0.64 - 0.79)** | **<.001** |
| **Carbamazepine** | 0.74 (0.54 – 1.01) | .090 | 1.18 (0.76 – 1.84) | .571 | **0.55 (0.39 – 0.77)** | **.001** |
| **Quetiapine** | **0.84 (0.77 – 0.92)** | **<.001** | 0.84 (0.73 – 0.98) | .051 | **0.83 (0.74 – 0.94)** | **.004** |
| **Olanzapine** | **0.77 (0.70 – 0.83)** | **<.001** | **0.76 (0.66 – 0.87)** | **<.001** | **0.76 (0.69 – 0.84)** | **<.001** |
| **Aripiprazole** | **0.77 (0.67 – 0.89)** | **<.001** | 0.81 (0.60 – 1.07) | .210 | **0.75 (0.64 – 0.88)** | **<.001** |
| **Risperidone (oral)** | 0.98 (0.81 – 1.19) | .868 | 0.92 (0.65 – 1.30) | .730 | 1.00 (0.79 – 1.26) | .981 |
| **Risperidone (LAI)** | 0.48 (0.24 – 0.96) | .062 | 0.39 (0.14 – 1.11) | .148 | 0.54 (0.23 – 1.29) | .249 |
| **Paliperidone (oral)** | 0.57 (0.18 – 1.86) | .439 | 0.73 (0.11 – 4.90) | .832 | 0.57 (0.15 – 2.22) | .518 |
| **Paliperidone (LAI)** | 1.21 (0.55 – 2.68) | .701 | 0.73 (0.33 – 1.64) | .570 | nil estimate | – |
| **Ziprasidone** | 0.88 (0.56 – 1.38) | .644 | 0.45 (0.17 – 1.21) | .176 | 1.04 (0.67 – 1.63) | .904 |
| **Clozapine** | 0.67 (0.37 – 1.23) | .272 | 0.39 (0.18 – 0.86) | .460 | 0.99 (0.40 – 2.44) | .981 |
| **Events, n** | 22,681 |  | 8,338 |  | 14,343 |  |

Abbreviations: *n*=number of; CI = confidence interval; LAI = long-acting injectable

^a^ Adjusted for: each other drug; cumulative number of switches per drug; polypharmacy; number of previous bipolar rehospitalizations; age, subdiagnosis, and history of substance- and alcohol- use disorder comorbidity at past admission; duration of past admission; year of last discharge and refill, respectively.

^b^ Corrected for multiple comparisons with the Benjamini-Hochberg procedure

# Table e6. Sensitivity analysis. Alternative threshold of four months (122 days) between dispensed prescriptions to define maintenance therapy. Adjusted associations between the time spent on- versus off- maintenance therapy with bipolar disorder rehospitalization. Within-individual design; extended Cox regression model.

| **Maintenance drug** | **Total cohort (n=15,988)** |  | **Males (n=6,400)** |  | **Female s (n=9,588)** |  |
| --- | --- | --- | --- | --- | --- | --- |
|  | **Hazard ratio^a^ (95% CI)** | **P-value^b^** | **Hazard ratio^a^ (95% CI)** | **P-value^b^** | **Hazard ratio^a^ (95% CI)** | **P-value^b^** |
| **Lithium** | **0.67 (0.62 – 0.73)** | **<.001** | **0.66 (0.58 – 0.74)** | **<.001** | **0.67 (0.60 – 0.74)** | **<.001** |
| **Valproate** | **0.73 (0.66 – 0.82)** | **<.001** | **0.80 (0.69 – 0.93)** | **.005** | **0.68 (0.58 – 0.80)** | **<.001** |
| **Lamotrigine** | **0.69 (0.64 – 0.76)** | **<.001** | **0.71 (0.61 – 0.82)** | **<.001** | **0.67 (0.61 – 0.75)** | **<.001** |
| **Carbamazepine** | 0.75 (0.53 – 1.06) | .145 | 1.40 (0.82 – 2.42) | .256 | **0.50 (0.36 – 0.70)** | **<.001** |
| **Quetiapine** | **0.73 (0.67 – 0.81)** | **<.001** | **0.77 (0.66 – 0.90)** | **.002** | **0.71 (0.63 – 0.81)** | **<.001** |
| **Olanzapine** | **0.77 (0.71 – 0.84)** | **<.001** | **0.77 (0.67 – 0.88)** | **<.001** | **0.77 (0.70 – 0.86)** | **<.001** |
| **Aripiprazole** | **0.71 (0.60 – 0.83)** | **<.001** | **0.67 (0.50 – 0.90)** | **.013** | **0.71 (0.58 – 0.86)** | **<.001** |
| **Risperidone (oral)** | 0.84 (0.69 – 1.02) | .107 | 0.68 (0.46 – 1.01) | .075 | 0.89 (0.71 – 1.11) | .365 |
| **Risperidone (LAI)** | **0.31 (0.12 – 0.82)** | **.031** | 0.57 (0.12 – 2.74) | .534 | **0.20 (0.11 – 0.36)** | **<.001** |
| **Paliperidone (oral)** | 0.86 (0.15 – 4.81) | .888 | nil estimate | – | nil estimate | – |
| **Paliperidone (LAI)** | 0.65 (0.23 – 1.84) | .469 | nil estimate | – | nil estimate | – |
| **Ziprasidone** | 0.69 (0.40 – 1.20) | .240 | **0.18 (0.07 – 0.49)** | **.002** | 0.87 (0.53 – 1.43) | .636 |
| **Clozapine** | **0.37 (0.15 – 0.88)** | **.041** | nil estimate | – | 0.56 (0.23 – 1.32) | .235 |
| **Events, n** | 22,681 |  | 8,338 |  | 14,343 |  |

Abbreviations: *n*=number of; CI = confidence interval; LAI = long-acting injectable

^a^ Adjusted for: each other drug; cumulative number of switches per drug; polypharmacy; number of previous bipolar rehospitalizations; age, subdiagnosis, and history of substance- and alcohol- use disorder comorbidity at past admission; duration of past admission; year of last discharge and refill, respectively.

^b^ Corrected for multiple comparisons with the Benjamini-Hochberg procedure

# Table e7. Sensitivity analysis. Maintenance periods extended by 30 days. Adjusted associations between the time spent on- versus off- maintenance therapy with bipolar disorder rehospitalization. Within-individual design; extended Cox regression model.

| **Maintenance drug** | **Total cohort (n=15,988)** |  | **Males (n=6,400)** |  | **Female s (n=9,588)** |  |
| --- | --- | --- | --- | --- | --- | --- |
|  | **Hazard ratio^a^ (95% CI)** | **P-value^b^** | **Hazard ratio^a^ (95% CI)** | **P-value^b^** | **Hazard ratio^a^ (95% CI)** | **P-value^b^** |
| **Lithium** | **0.73 (0.68 – 0.79)** | **<.001** | **0.71 (0.63 – 0.80)** | **<.001** | **0.73 (0.67 – 0.80)** | **<.001** |
| **Valproate** | **0.81 (0.73 – 0.90)** | **<.001** | 0.87 (0.76 – 1.01) | .093 | **0.77 (0.66 – 0.89)** | **.001** |
| **Lamotrigine** | **0.75 (0.69 – 0.81)** | **<.001** | **0.78 (0.69 – 0.89)** | **<.001** | **0.72 (0.65 – 0.80)** | **<.001** |
| **Carbamazepine** | **0.66 (0.49 – 0.88)** | **.009** | 0.89 (0.58 – 1.37) | .657 | **0.57 (0.41 – 0.80)** | **.002** |
| **Quetiapine** | **0.81 (0.74 – 0.88)** | **<.001** | 0.86 (0.73 – 1.00) | .078 | **0.78 (0.69 – 0.86)** | **<.001** |
| **Olanzapine** | **0.82 (0.75 – 0.89)** | **<.001** | **0.82 (0.72 – 0.94)** | **.011** | **0.80 (0.72 – 0.89)** | **<.001** |
| **Aripiprazole** | **0.75 (0.63 – 0.88)** | .**001** | 0.73 (0.54 – 0.98) | .068 | **0.76 (0.62 – 0.93)** | **.011** |
| **Risperidone (oral)** | 0.91 (0.77 – 1.08) | .331 | 1.09 (0.82 – 1.44) | .637 | 0.85 (0.70 – 1.04) | .159 |
| **Risperidone (LAI)** | **0.31 (0.12 – 0.82)** | **.032** | 0.22 (0.02 – 2.79) | .298 | **0.37 (0.19 – 0.73)** | **.006** |
| **Paliperidone (oral)** | 0.88 (0.16 – 4.86) | .910 | 1.15 (0.12 – 11.10) | .926 | nil estimate | – |
| **Paliperidone (LAI)** | 0.88 (0.36 – 2.12) | .812 | nil estimate | – | nil estimate | – |
| **Ziprasidone** | 0.75 (0.48 – 1.16) | .252 | **0.43 (0.27 – 0.67)** | **<.001** | 0.96 (0.63 – 1.47) | .875 |
| **Clozapine** | 0.76 (0.41 – 1.41) | .441 | 0.54 (0.20 – 1.47) | .288 | 0.89 (0.52 – 1.53) | .725 |
| **Events, n** | 22,681 |  | 8,338 |  | 14,343 |  |

Abbreviations: *n*=number of; CI = confidence interval; LAI = long-acting injectable

^a^ Adjusted for: each other drug; cumulative number of switches per drug; polypharmacy; number of previous bipolar rehospitalizations; age, subdiagnosis, and history of substance- and alcohol- use disorder comorbidity at past admission; duration of past admission; year of last discharge and refill, respectively.

^b^ Corrected for multiple comparisons with the Benjamini-Hochberg procedure

# Table e8. Sensitivity analysis. Outcome restricted to rehospitalizations for manic, depressive or mixed episodes^a^. Adjusted associations between the time spent on- versus off- maintenance therapy with bipolar disorder rehospitalization. Within-individual design; extended Cox regression model.

| **Maintenance drug** | **Total cohort (n=11,252)** |  | **Males (n=4,502)** |  | **Female s (n=6,750)** |  |
| --- | --- | --- | --- | --- | --- | --- |
|  | **Hazard ratio^b^ (95% CI)** | **P-value^c^** | **Hazard ratio^b^ (95% CI)** | **P-value^c^** | **Hazard ratio^b^ (95% CI)** | **P-value^c^** |
| **Lithium** | **0.63 (0.56 – 0.71)** | **<.001** | **0.57 (0.47 – 0.68)** | **<.001** | **0.65 (0.56 – 0.75)** | **<.001** |
| **Valproate** | **0.73 (0.61 – 0.88)** | **.002** | 0.75 (0.57 – 0.99) | .075 | **0.71 (0.57 – 0.89)** | **.006** |
| **Lamotrigine** | **0.68 (0.60 – 0.78)** | **<.001** | **0.72 (0.57 – 0.91)** | **.013** | **0.65 (0.56 – 0.76)** | **<.001** |
| **Carbamazepine** | 0.59 (0.35 – 0.98) | .063 | 0.73 (0.39 – 1.36) | .400 | 0.50 (0.25 – 1.01) | .081 |
| **Quetiapine** | **0.80 (0.70 – 0.92)** | **.003** | 0.87 (0.68 – 1.11) | .334 | **0.77 (0.65 – 0.90)** | **.003** |
| **Olanzapine** | **0.72 (0.63 – 0.82)** | **<.001** | **0.69 (0.55 – 0.86)** | **.003** | **0.73 (0.62 – 0.85)** | **<.001** |
| **Aripiprazole** | **0.63 (0.49 – 0.82)** | **.001** | **0.54 (0.33 – 0.87)** | .**026** | **0.68 (0.50 – 0.91)** | **.018** |
| **Risperidone (oral)** | 0.95 (0.70 – 1.30) | .815 | 1.12 (0.66 – 1.91) | .708 | 0.89 (0.62 – 1.26) | .610 |
| **Risperidone (LAI)** | 0.53 (0.21 – 1.38) | .261 | 0.58 (0.18 – 1.81) | .400 | 0.43 (0.12 – 1.46) | .246 |
| **Paliperidone (oral)** | nil estimate | – | nil estimate | – | nil estimate | – |
| **Paliperidone (LAI)** | **0.27 (0.09 – 0.84)** | **.039** | nil estimate | – | **0.21 (0.09 – 0.53)** | **.002** |
| **Ziprasidone** | 0.64 (0.31 – 1.32) | .279 | nil estimate | – | 0.90 (0.45 – 1.83) | .832 |
| **Clozapine** | **0.40 (0.19 – 0.85)** | **.029** | 0.37 (0.13 – 1.05) | .102 | **0.43 (0.23 – 0.79)** | **.013** |
| **Events, n** | 10,360 |  | 3,796 |  | 6,564 |  |

Abbreviations: *n*=number of; CI = confidence interval; LAI = long-acting injectable

^a^ ICD-10 diagnostic clusters used to define episodes; manic (F30x, F31.0-F31.2), depressive (F31.3-F31.5, F32x, F33x) or mixed (F31.6).

^b^ Adjusted for: each other drug; cumulative number of switches per drug; polypharmacy; number of previous bipolar rehospitalizations; age, subdiagnosis (recoded as a three-level factor as other diagnostic levels were excluded), and history of substance- and alcohol- use disorder comorbidity at past admission; duration of past admission; year of last discharge and refill, respectively.

^c^ Corrected for multiple comparisons with the Benjamini-Hochberg procedure
